# Application of Box-Behnken design to optimize the phosphorus removal from industrial wastewaters using magnetic nanoparticles

**DOI:** 10.1007/s11356-025-36152-6

**Published:** 2025-02-28

**Authors:** Celso E. D. Cardoso, Joana C. Almeida, João Rocha, Eduarda Pereira

**Affiliations:** 1 Chemistry Department and CICECO-Aveiro Institute of Materials, University of Aveiro, Campus de Santiago, 3810-193 Aveiro, Portugal; 2 Chemistry Department and LAQV-REQUIMTE, University of Aveiro, Campus de Santiago, 3810-193 Aveiro, Portugal

**Keywords:** Cobalt ferrite nanoparticles, Phosphate, Adsorption, Eutrophication, Box-Behnken design, Response surface methodology, Design of experiments

## Abstract

Phosphorus is essential for all living organisms and limits aquatic plant growth. Pulp mill effluents, particularly from *Eucalyptus* bleached kraft pulp mills, contain phosphorus concentrations that vary with operational conditions. This variability poses challenges for effective treatment and phosphorus removal. However, uncontrolled release of phosphorus-rich wastewaters causes eutrophication. This study focuses on optimizing phosphorus removal from such effluents using cobalt ferrite nanoparticles, with an emphasis on process optimization to address this variability. Minimizing phosphorus concentrations is crucial in wastewater engineering and surface water management. By employing design of experiments and response surface methodology, we aim to fine-tune the phosphorous removal process and pinpoint the key factors with the most significant impact. Optimal conditions for achieving over 90% removal from an effluent with 5 mg P/L were identified as a sorbent dose greater than 1.3 g/L and a pH range between 5 and 7, all within a contact time of only 15 min. For a contact time of 1 and 24 h, the conditions adjust to a sorbent dose greater than 0.97 and 0.83 g/L, respectively, with the pH range remaining the same. Our results highlight the effectiveness of cobalt ferrite nanoparticles as sorbents in the removal of phosphorus for water treatment purposes. This approach presents a sustainable and proficient strategy for phosphorus recovery from pulp mill effluents, thereby lessening environmental repercussions and offering a valuable resource for future use. This contributes to the maintenance of water quality and ecosystem preservation.

**Supplementary Information:**

The online version contains supplementary material available at 10.1007/s11356-025-36152-6.

## Introduction

Environmental concerns emerged after the Industrial Revolution due to untreated industrial effluents contaminating water bodies with hazardous pollutants (Chowdhary et al. 2020; Garg et al. 2022). Growing populations and urban migration, driven by climate change and the pursuit of better living standards (United Nations Environment Programme 2019), are expected to increase these discharges, intensifying water stress and degrading aquatic resources both quantitatively (e.g., aquifer depletion) and qualitatively (e.g., eutrophication, organic matter levels). Excessive phosphorus (P), from fertilizers, wastewater, and industrial byproducts, leads to eutrophication, harming water quality and aquatic life (Ngatia and Taylor 2019; Penn and Bowen 2017; World Water Quality Alliance 2021). Controlling eutrophication involves reducing nutrient concentrations, especially P. Therefore, regulating P discharge is vital for preserving water quality, as required by European Union environmental policies (European Commission 2014).

In the pulp and paper industry, over 90% of the P in pulp production comes from wood, affecting the final effluent’s P levels. *Eucalyptus globulus* wood, used in some production units, shows higher initial P levels due to the specific edaphic-climatic and soil characteristics of this species in the Iberian Peninsula (European Commission 2014). Current Best Available Techniques (BAT) for this sector set P emission levels for bleached kraft pulp mills at 0.01 to 0.03 kg/ADt (air-dried tonnes). However, an exception applies to facilities processing *Eucalyptus* wood due to its naturally higher P content, allowing for emissions of 0.02–0.11 kg/ADt (European Commission 2014). This implies P levels must be below 1.2 mg/L for mills generating up to 50 m^3^/ADt wastewater annually, and less than 4.4 mg/L for *Eucalyptus* mills (European Commission 2014). While these BAT limitations exist, achieving them might not be sufficient to prevent eutrophication, as research suggests a significant risk at P levels exceeding 100 µg/L (Kumar et al. 2019). As environmental regulations tighten, the industry must anticipate stricter controls and invest in innovative methods to minimize nutrient pollution.

Effluent treatment processes containing P are categorized into physical, biological, and chemical methods. Primary treatment includes physico-chemical processes like equalization, neutralization, and sedimentation to prevent system overload. Secondary treatment uses microorganisms for treatment, followed by a clarification phase to separate solids and biomass from effluents (European Commission 2014). Studies revealed that primary and secondary treatments can remove only 5–15% and 30–50% of nutrients, respectively. Tertiary treatment, crucial for removing most P, is especially important in bleached kraft pulp mills processing *Eucalyptus* (Penn and Bowen 2017; WHO and Commission 2002). It involves advanced techniques like flocculation, precipitation, and filtration to reduce P levels. Chemical precipitation is a common method for P removal, using compounds such as iron and aluminum salts due to their cost-effectiveness (European Commission 2014). An alternative approach involves the use of calcium, particularly calcium hydroxide, which is also widely used industrially (Machado et al. 2005). Other materials like calcite (Bańkowska-Sobczak 2021; Liu et al. 2012), dolomite (Ayoub et al. 2019), aragonite (Khan et al. 2021; Wang et al. 2021), and biominerals in eggshells and shells (Lee et al. 2022; Quisperima et al. 2022) have potential applications. Some studies highlight the potential of calcite to remove P under strongly acidic and basic conditions (Liu et al. 2012). Magnesium salts are another option, but their lower performance and specific reaction conditions limit their use (European Commission 2014; Velusamy et al. 2021; Wu et al. 2020). The main drawback of chemical precipitation is the generation of excessive sludge with high moisture content, classified as hazardous waste (Wijewardena 2018). The increasing strictness of P regulations challenges traditional methods like chemical precipitation and biological P removal due to their limitations in achieving low P concentrations (Wu et al. 2020). Despite these existing methods, there remains a significant gap in developing high-efficiency, low-waste treatment solutions that are specifically tailored to the unique challenges of P removal in *Eucalyptus* kraft pulp mill effluents.

Sorption emerges as a promising alternative owing to its efficiency, potential for sorbent regeneration, and minimal waste generation. A variety of sorbent materials are available, with lanthanum-based materials being the most reported for P removal due to their strong affinity with phosphates (Kumar et al. 2019). Layered double hydroxides (LDHs) based on Mg/Al, Zn/Al, and Ca/Al have shown potential (Liu et al. 2019; Loganathan et al. 2014), with their specific properties being designed and synthesized to meet operating conditions. Nanomaterials with high surface area are attracting interest for P removal (Kassem et al. 2022; Kumar et al. 2021; Suazo-Hernández et al. 2023; Tu et al. 2015). Examples include double zero-valent iron (Fe⁰) (Liu et al. 2018), nanoparticles (NPs) (Kumar et al. 2018), and mixtures of iron oxides, titanium oxides, and silicon oxides (Loganathan et al. 2014; Wu et al. 2020). Hydrated zirconium oxide, manganese oxide, and zinc ferrite have also been explored (Ahmed et al. 2019; Liu et al. 2018). Surface modification with appropriate organic materials can further enhance P selectivity (Loganathan et al. 2014). Sorption mechanisms like adsorption or co-precipitation using metal-based sorbents often involve strong P-nanomaterial bonds (Loganathan et al. 2014; Wu et al. 2020). Magnetic nanomaterials are gaining attention as potential sorbents due to their small size, cost efficiency, strong reactivity, large surface area, and numerous active sites. Chen et al. (2020) reported a magnetic lanthanum composite, La(OH)_3_-modified magnetic cobalt ferrite nanocomposite (La2-CF), with an adsorption capacity of 104.01 mg P/g. Tu and You (2014) developed a nano-bimetal ferrite (CuFe_2_O_4_) for P removal. The results reveal a rapid (120 min) P removal, from 9.9 to 99.9%, when the solution pH decreased from 9.06 to 2.64. The maximum P adsorption capacity was found to be 13.5 mg/g at pH 2.64. Magnetic properties allow easy separation from water, eliminating the need for conventional and labor-intensive purification methods such as filtration and sedimentation. Despite numerous nanoadsorbents reported for P removal, spinel ferrites have shown significant promise due to their magnetic properties, large surface area, and high adsorption capacity, but their performance in complex effluents, particularly from *Eucalyptus*-based pulp mills, remains underexplored. Existing research often uses unrealistic and simplified conditions.

The present study investigates the potential of cobalt ferrite (CoFe_2_O_4_) NPs for P removal from a *Eucalyptus* bleached kraft pulp mill effluent using design of experiments (DoE) and response surface methodology (RSM) approach. DoE is employed to recognize the relationship between variables and responses, identifying key factors affecting the response (Witek-Krowiak et al. 2014). RSM uses these experimental results to create a model that predicts the optimal conditions for P removal. The effectiveness of RSM depends on the quality of data provided by DoE. The Box-Behnken design (BBD) was the model selected to perform DoE and RSM. BBD is an incomplete factorial design with three levels, proficient in modeling linear, quadratic, and interaction effects using second-order polynomials (Witek-Krowiak et al. 2014). It requires fewer experiments and allows to know the optimum removal conditions with high precision. Nevertheless, BBD has regions of low prediction quality at the extreme (highest or lowest values) points, and this needs to be in consideration in the planning of the experimental design. This tool offers significant advantages for the industrial sector as it allows for rapid prediction and adaptation to the daily changing conditions of effluents.

To optimize the process of P sorption, CoFe_2_O_4_ NPs were synthesized, characterized, and subsequently evaluated as a promising adsorbent for P removal under varying conditions of pH, sorbent dose, and initial P concentration. Furthermore, an additional study was conducted to assess the impact of temperature in the optimal conditions. The gaps in existing research that this study addresses are as follows: (1) the optimization of phosphorus removal for real-world industrial applications, particularly in kraft pulp mill effluents, as opposed to synthetic or model waters used in previous studies; (2) the development of a comprehensive process optimization framework using RSM to handle the inherent variability of pulp mill effluents in real time.

This work is focused on significantly reducing P levels in pulp mill effluents, thereby aligning with environmental agency standards on P emissions and curtailing the potential for eutrophication. It represents a significant advancement in sustainable wastewater treatment for the pulp industry by combining magnetic nanoparticle technology with process optimization using statistical tools.

## Experimental section

### Materials and reagents

All reagents used in this study were obtained from certified suppliers and used without additional purification. Potassium hydroxide (KOH, > 98%), potassium nitrate (KNO_3_, > 99%), ferrous sulfate heptahydrate (FeSO_4_^.^7H_2_O, > 99%), cobalt chloride hexahydrate (CoCl_2_^.^6H_2_O, > 98%), and potassium dihydrogen phosphate (KH_2_PO_4_, > 99%) were obtained from Chem-lab NV. Nitric acid (HNO_3_, 65%) and sodium hydroxide (NaOH, > 98%) were obtained from Merck and Pronolab, respectively. The ultrapure water (18 MΩ cm) used was produced by a Millipore Integral 10 system. All glassware was previously cleaned with nitric acid (HNO_3_ 25% v/v) sourced from Merck, Suprapur® 65%, for a minimum duration of 24 h. Following this, a rinse with ultrapure water (Milli-Q water, 18 MΩ/cm) was performed to ensure complete removal of the acid.

Sorption experiments were performed using an industrial wastewater collected from a *Eucalyptus* bleached kraft pulp mill. This wastewater refers to the effluent obtained after biological treatment (secondary treatment) of a wastewater treatment plant (WWTP). Typically, the effluent exhibits a complex and variable matrix. The pH range lies between 6.2 and 7.8, the conductivity between 3.4 and 4.1 mS/cm, and the total suspended solids range from 10 to 18 mg/L. Furthermore, it contains 9 mg/L of nitrogen, 1070 mg/L of sulfate, Na levels ranging from 623 to 2009 mg/L, Ca levels from 102 to 680 mg/L, K levels from 33 to 121 mg/L, and Mg levels from 7.3 to 15 mg/L. Trace elements were also detected, including Al, Fe, Mn, As, Cu, Li, Zn, and Sr, all measured in micrograms per liter (µg/L). The P concentration levels are between 2 and 20 mg P/L.

### Preparation of cobalt ferrite nanoparticles

Cobalt ferrite NPs with an average size of 50 nm were synthesized by oxidative hydrolysis of iron(II) sulfate in alkaline conditions, based on the procedure described in Tavares et al. (2020). Briefly, ultrapure water was first subjected to a deoxygenation process under N_2_ nitrogen gas with intense stirring for a duration of 2 h. Following this, 15.20 g (34 mmol) of KOH and 12.16 g (15 mmol) of KNO_3_ were introduced into 200 mL of deoxygenated water in a 500-mL round flask. This blend was then heated at 60 °C, in a nitrogen environment and mechanically stirred at 500 rpm.

Upon achieving complete dissolution, an aqueous solution of 200 mL comprising FeSO_4_·7H_2_O (24.56 g, 11 mmol) and CoCl_2_·6H_2_O (11.52 g, 6 mmol) was incrementally added dropwise to the bend while the stirring speed was ramped up to 700 rpm. After complete addition of mixture solution, the resulting solution presented a dark green color. The solution was allowed to react for 30 min. Next, the round flask was transferred to a hot oil bath at 90 °C under a nitrogen environment without any stirring for 4 h. Finally, the resultant dark brown powder was washed multiple times with deoxygenated water and ethanol. Post-washing, the particles were dried by allowing the solvent to evaporate in an oven set at 60 °C.

### Structural and chemical characterization of cobalt ferrite nanoparticles

The particle morphology was investigated using transmission electron microscopy (TEM) (JEOL, 2200FS, operating at 200 kV). A TEM specimen was prepared by directly depositing an aliquot of an ethanolic suspension of NPs onto a copper grid coated with a carbon film. The solvent was allowed to evaporate at room temperature, leaving behind a thin film of NPs on the grid. The zeta potential of the colloidal CoFe_2_O_4_ NP dispersion was measured using a Zetasizer Nano ZS (Malvern Instruments). The pH of the colloidal dispersion was varied between 4 and 8 using aqueous solutions of either sodium hydroxide (NaOH) or nitric acid (HNO_3_). The temperature was maintained at 25 °C, and three replicate measurements of zeta potential were performed for each sample. The phase purity of the powdered CoFe_2_O_4_ NPs was determined using X-ray diffraction (XRD) analysis. XRD patterns were obtained using an Empyrean PANalytical diffractometer equipped with Cu Kα1,2 X-rays (λ1 = 1.54060 Å; λ2 = 1.54443 Å). The diffraction patterns were recorded in continuous mode with a step size of 0.026°, covering the 15 ≤ 2θ ≤ 95° range. The surface chemical composition of the CoFe_2_O_4_ NPs was investigated using Fourier transform infrared (FT-IR) spectroscopy. FT-IR spectra were recorded in the range of 3800–400 cm^−1^ using a Bruker Tensor 27 spectrophotometer. Equipped with an attenuated total reflectance (ATR) accessory, after 256 scans with resolution of 4 cm^−1^. The Brunauer-Emmet-Teller (BET) was measured using a Micromeritics Gemini 2380 automated surface area analyzer, employing nitrogen adsorption–desorption techniques. For the investigation of magnetic properties, hysteresis loop measurements were conducted with a vibrating sample magnetometer (VSM) at room temperature (27 °C). The magnetization values were normalized to the total mass of the sample.

### Measurement of phosphorus concentration

The quantification of P in solution was performed using inductively coupled plasma-optical emission spectrometry (ICP-OES) on a spectrometer from Horiba Jobin Yvon Activa M (in a radial configuration), which was equipped with a nebulizer from Burgener MiraMist. Calibration curves were constructed with five standards ranging from 0.1 to 26 mg/L, prepared by diluting commercially available certified P stock solutions into acidified water (1% HNO_3_ v/v). Only calibration curves with a correlation coefficient exceeding 0.9995 were accepted. The limit of quantification was considered as the lowest standard of calibration curve (0.1 mg/L). The coefficient of variation between replicate sample measurements (at least three replicates) was consistently below 5%.

To determine the amount of P retained by the sorbent material per unit of mass, *q* (mg/g), given the assumption that all the P removed from the solution was adhered to the sorbent, it was used the following Eq. (1):

1 $$q=\frac{{(C}_{0}-{C}_{t})}{m}*V$$

where ${C}_{0}$ (mg/L) is the initial concentration of P in solution, ${C}_{t}$ (mg/L) is the concentration of P at time $t$, $m$ is the mass of sorbent (g), and $V$ is the solution volume (L).

The recovery efficiency, *R* (%), for P in CoFe_2_O_4_ NPs was calculated as follows (Eq. (2)):

2 $$\text{Removal }({\%})=\frac{{(C}_{0}-{C}_{t})}{{C}_{0}}*100$$

### Planification of the design of experiments and response surface methodology essays

As previously mentioned, multivariate statistical techniques such as DoE and RSM are an alternative and improvement to the conventional “one variable at a time” method, which is more suitable for optimizing the adsorption process. RSM uses the experimental results generated by DoE to fit a mathematical model that predicts the response as a function of the factors studied (Witek-Krowiak et al. 2014). These methodologies facilitate the optimization of the adsorption process, leading to a substantial decrease in the number of required experiments and a reduction of associated cost. Generally, the application of these methodologies for optimization follows a procedure: (i) identification of the problem and the objective to achieve, (ii) selection of the response variable, (iii) selection of independent variables and work levels, (iv) selection of experimental design strategy, (v) realization of experimental test and fitting the model equation to experimental data, (vi) obtaining response graphs and verification of the model, (vii) determination of optimal conditions (Witek-Krowiak et al. 2014).

In this study, the objective of the experimental design is to achieve a significant reduction in the P concentration in the final effluent from pulp mills. The response variable for that purpose is the percentage of P removal. The chosen independent variables include the initial P concentration (mg/L), the pH of the medium, and the sorbent dose (g/L). In addition to these factors, the contact time between the sorbent and the effluent being treated also impacts the percentage of P removal. Consequently, three contact times were defined as follows: 15 min, 1 h, and 24 h. The working levels of the experimental parameters, as illustrated in Table 1, were defined based on the conditions of the effluent. These conditions can vary significantly throughout the year, depending on the wood P content that is loaded, its origin, the species of *Eucalyptus* added to the process, and whether purges are performed or not. All these factors contribute to the dynamic nature of the effluent’s composition. Consequently, a wide range of different parameters was chosen to cover the variability of effluent conditions and to provide a better process optimization. The BBD is the most suitable method for optimizing the adsorption process given the specific conditions mentioned. It significantly reduces the number of experiments required compared to factorial designs, leading to cost savings and increased time efficiency. It provides precise estimates of linear and quadratic effects, enhancing understanding of the system and identification of optimal removal conditions. Furthermore, BBD facilitates the construction of response surfaces, providing visual insights into process behavior. The Minitab 19 program was used to carry out this study and to generate combinations of different conditions. Each variable was placed as three equidistant values (− 1; 0; 1), as presented in Table 1. The impact of these variables was evaluated on the performance of CoFe_2_O_4_ NPs, for the P removal (%) and the extent of the accumulation (P concentrations in NPs, *q*). To evaluate the precision, three replicates in the central point were carried out. By using replicated center points, it is possible to determine how well the model fits the data and to identify potential sources of lack of fit. This is crucial in accurately predicting the response of the system and optimizing the process parameters to achieve the desired outcome. The experiments generated by BBD are shown in Table 1SI (in Online Resource). Different amounts of NPs (0.2, 1.35, and 2.5 g/L) were placed in contact with three different P concentrations (2, 14, and 26 mg/L). Additionally, pH was adjusted to 4, 7, and 10 with HNO_3_ 2% (v/v) and NaOH (1 and 10 mol/L). Solution samples were taken in predetermined periods (0 and 15 min, 1 h, and 24 h) after NP addition (0 h), immediately magnetic separated and stored at 4 °C for further quantification of P. Control solutions (wastewater in absence of NPs) were always run in parallel with the assays to evaluate potential experimental losses of P.

**Table 1:** Experimental conditions for the three different factors studied and the three work levels

Variable	Level		
	− 1	0	1
pH	4	7	10
Dose of sorbent (g/L)	0.20	1.35	2.50
Initial concentration of P (mg/L)	2	14	26

The efficiency of P removal by CoFe_2_O_4_ NPs was further investigated in the wastewater at different temperatures (20, 40, 60 °C). The selected operational parameters for this study, which include P initial concentration, sorbent dose, and pH, were set to match the “optimal conditions” determined by RSM. The results were obtained using the software Design-Expert version 13 (Stat-Ease Inc.) and Minitab Statistical Software version 19.

## Results and discussion

### Cobalt ferrite nanoparticle characteristics

**Fig. 1:**
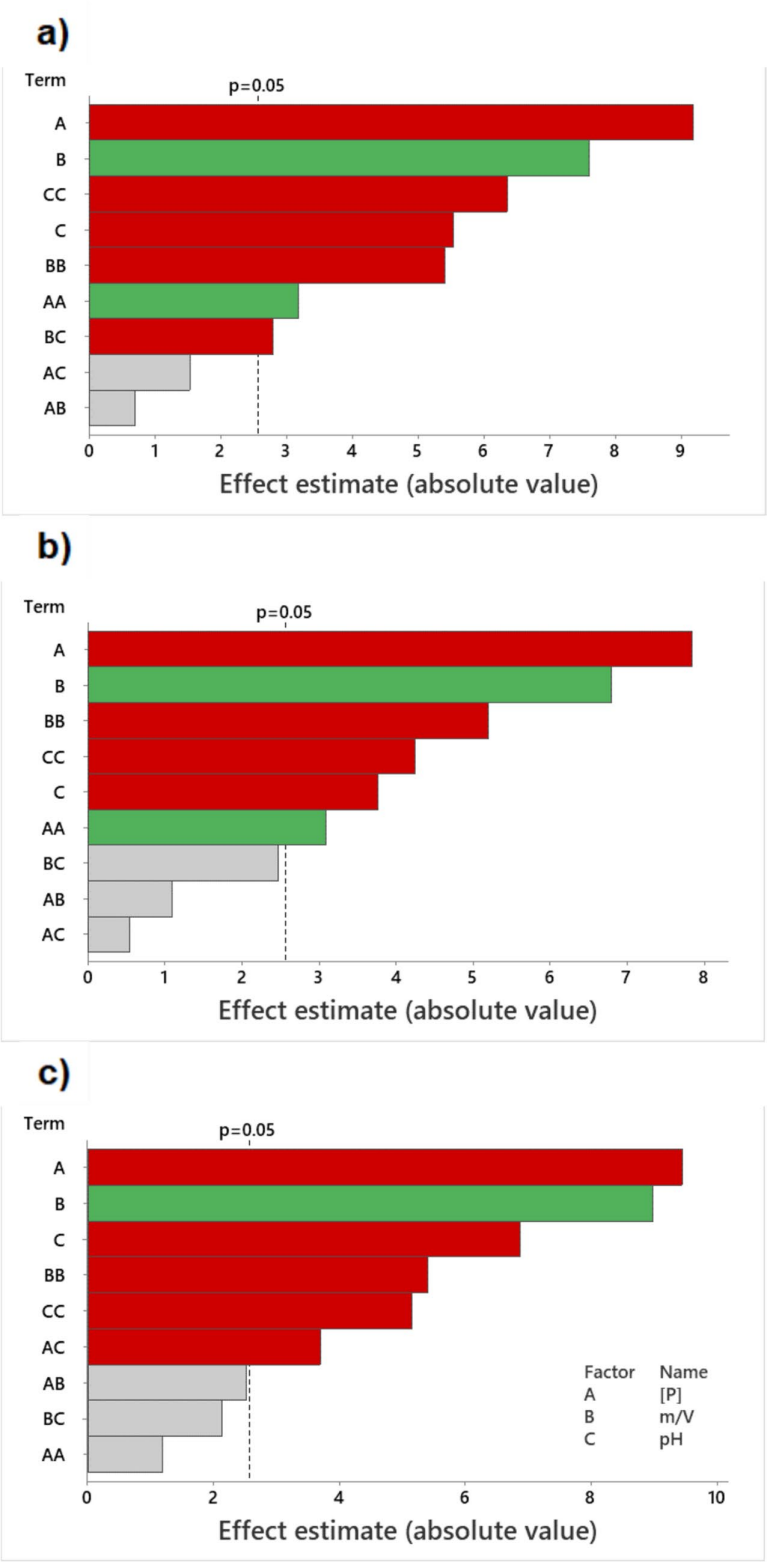
Pareto chart with the effects of the factors in the response studied (removal (%)) at (**a**) 15 min, (**b**) 1 h, and (**c**) 24 h of exposure. In the figure: A represents the initial P concentration (mg/L), B is the sorbent dose (g/L), and C is the pH. Factors with values below the dashed line are not significant

To confirm the crystalline phase of obtained CoFe_2_O_4_, powder X-ray diffractogram was acquired (Fig. 1aSI, in Online Resource) and the peaks corresponding to the crystalline planes (1 1 1), (2 2 0), (3 1 1), (4 0 0), (4 2 2), (5 1 1), (4 4 0), and (533) at º2θ equal to 18.3, 30.1, 35.5, 43.1, 53.5, 57.1, 62.7, and 74.1 indexed. Comparison with database patterns confirmed the spinel ferrite structure for the CoFe_2_O_4_ NPs synthesized. ICP measurements afforded a Co:Fe molar ratio of 1.00:1.98, in accord with the stoichiometry of the CoFe_2_O_4_. The morphology of the CoFe_2_O_4_ NPs was investigated using TEM (Fig. 1bSI, in Online Resource). The particles have a spherical shape and an average size of 72.8 nm, varying between 47 and 99 nm. Figure 1cSI (in Online Resource) depicts the zeta potential of CoFe_2_O_4_ NPs as a function of solution pH, and PZC of 6.3. The FTIR spectrum of CoFe_2_O_4_ NPs (Fig. 1dSI, in Online Resource) exhibits a broad band in the 3642–2860 cm^−1^ spectral range attributed to the ν(OH), and the bending vibration δ(H–O-H) at 1632 cm^−1^, indicating the presence of molecular water adsorbed at the surface or incorporated into the crystalline lattice (Almeida et al. 2024; Tavares et al. 2020). Additionally, the peak at 1105 cm^−1^ is due to metal-OH and metal-OH_2_ bonds (Almeida et al. 2024), which reflect the sorption of water on the oxide. The peak at 564 cm^−1^ is due to metal–oxygen stretching vibration (Tavares et al. 2020). The NPs exhibit a BET surface area of 33.3 m^2^/g, a pore volume of 0.07 cm^3^/g, and an average pore diameter of 9.2 nm. These results are consistent with previous studies (Tavares et al. 2020). Coercivity and saturation magnetization values obtained from the hysteresis loops were 5.4 Oe and 83 emu/g, respectively. CoFe_2_O_4_ NPs demonstrate reusability due to their low coercivity, which allows for easy demagnetization. Furthermore, their high magnetic susceptibility plays a crucial role in their response to an external magnetic field. The saturation magnetization observed is closely aligned with that of bulk magnetite (92 emu/g).

### Influence of experimental variables studied with experiment design

The performed DoE is described in Table 1SI (in Online Resource) for 15 min, 1 h, and 24 h and the results of P removal (%) are presented in Table 2. The maximum P removal achieved by CoFe_2_O_4_ NPs for the three concentrations tested (2, 14, and 26 mg P/L) was observed on experiment 3 (initial P concentration of 2 mg/L, pH 7, and NP dosage of 2.5 g/L), and experiment 5 (initial P concentration of 2 mg/L, pH 4, and NP dosage of 1.35 g/L) with removal percentages of 97% after 15 min and 100% after 1 h and 24 h. Focusing only on the higher concentrations, 14 mg P/L and 26 mg P/L, the best P removal values were observed on experiment 10 (initial P concentration of 14 mg/L, pH 4, and NP dosage of 2.5 g/L) and on experiment 4 (initial P concentration of 26 mg/L, pH 7, and NP dosage of 2.5 g/L). The removal values achieved for experiment 10 were 61, 75, and 95% for 15 min, 1 h, and 24 h of contact time, respectively; and for experiment 4 were 62, 69, and 79% for 15 min, 1 h, and 24 h of contact time, respectively. The results showed that P removal (%) increased over time. However, extending the experimental time to 24 h did not offer any substantial benefits in terms of % removal compared to the cost of extending the time. For industrial applications, faster P sorption is more desirable. Longer contact times imply more expenses in energy, human resources, space, and technical feasibility, which make shorter sorption processes preferable. The higher P concentrations in the NPs (*q*) were obtained under the conditions of experiments 2 and 9 (initial P concentration of 26 mg/L, NP dosage of 0.2 g/L, and pH 7 and 4, respectively). Values observed for P at pH 7 were 12.71, 15.36, and 15.36 mg/g, and for P at pH 4 were 7.48, 8.62, and 19.58 mg/g, at 15 min, 1 h, and 24 h, respectively.

**Table 2:** Removal of P achieved by CoFe_2_O_4_ NPs, at 15 min, 1 h, and 24 h, under the different experimental conditions presented in Table 1SI (in Online Resource)

Experiment	Removal (%)			*q* (mg/g)		
	15 min	1 h	24 h	15 min	1 h	24 h
1	55	63	72	5.24	0.01	0.01
2	10	12	12	12.71	15.36	15.36
3	97	100	100	0.92	0.95	0.95
4	62	69	79	8.11	8.96	10.33
5	97	100	100	1.36	1.41	1.41
6	33	45	66	6.39	8.64	12.78
7	61	91	96	0.86	1.28	1.35
8	19	26	5	3.80	5.12	0.93
9	11	12	28	7.48	8.62	19.58
10	61	75	95	4.27	5.28	6.67
11	0	0	2	0.00	0.05	1.44
12	10	18	36	0.70	1.30	2.63
13	62	70	82	6.49	7.28	8.49
14	66	71	83	6.70	7.28	8.46
15	65	72	83	6.69	7.31	8.53

**Fig. 2:**
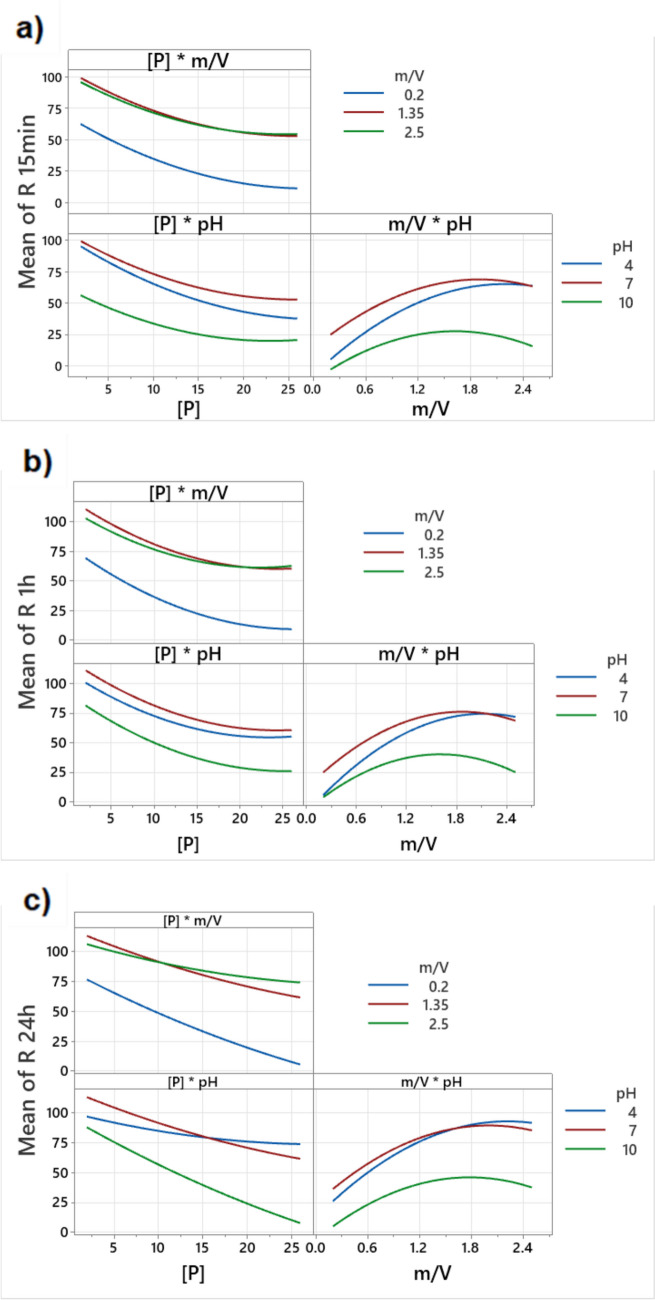
Interaction plots between the main effects affecting P removal after (**a**) 15 min, (**b**) 1 h, and (**c**) 24 h of contact time

The main factors influencing the P removal and their statistical importance in the response studied were identified by the results. Pareto charts, obtained with a confidence level of 95%, show the effect of the different factors (linear, interaction combinations, and quadratic effects) on the response (P removal (%) and *q*) (Figs. 1 and 2SI, in Online Resource). A, B, and C represent the concentration of P, dose of sorbent, and pH, respectively. The factors with a *p*-value < 0.05 are statistically significant for the response studied and are before the dashed vertical red line. The green bars indicate the factors that have a positive impact on the response, meaning that the response increases with the factor increase. The red bars indicate the factors that have a negative impact on the response, meaning that the response decreases with the factor increase. The grey bars indicate the factors that are not statistically significant for the response.

**Fig. 3:**
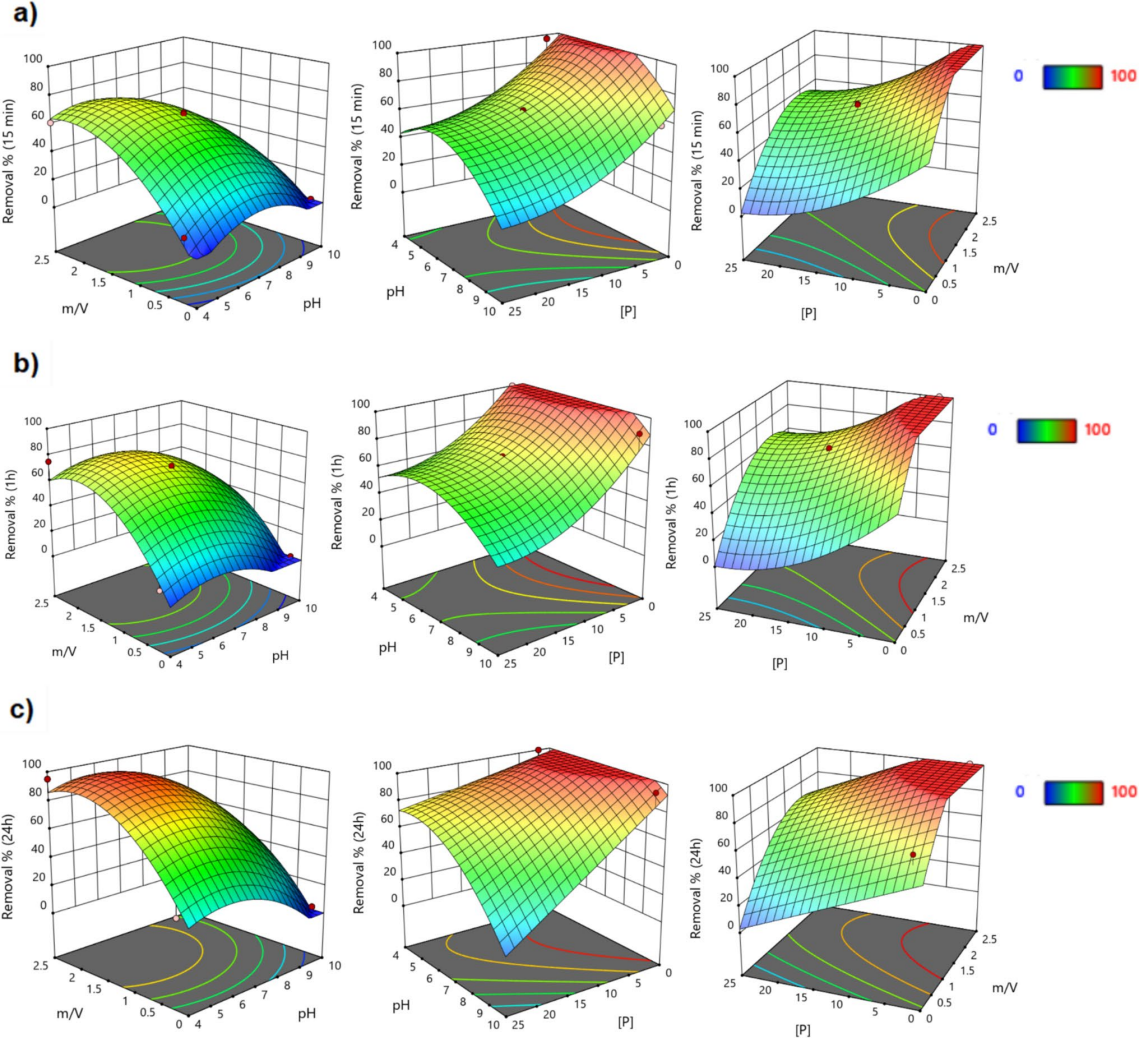
3D response surfaces obtained for the P removal with the reduced models after (**a**) 15 min, (**b**) 1 h, and (**c**) 24 h of exposure to CoFe_2_O_4_ NPs, as a function of the dose of sorbent and pH (on the left), pH and initial P concentration (in the middle), and initial P concentration and dose of sorbent (on the right). The variable not present in the plot is fixed at the central point (14 mg P/L, pH 7, and sorbent dose of 1.35 g/L)

Figure 1 shows that the most important effects of variables in the P removal are similar for the studied contact times. The linear effect of P concentration, having a negative impact on the response, is the most important, since with an increase of P concentration and maintaining the number of active sites, the removal (%) tends to decrease. In contrast, increasing the sorbent dose increases both the number of available active sites and the removal efficiency. This justifies the positive linear effect of sorbent dose as the second more important factor for P removal. Regarding pH, both linear and quadratic effects show a negative impact on the response, which is associated with the NP surface charge and the P species in solution. The effect of pH can be observed in more detail in Fig. 3SI (in Online Resource) that also shows the effects of P concentration, sorbent dose (m/V), and pH on the removal after 15 min, 1 h, and 24 h of contact time. Figure 2SI (in Online Resource) shows the most significant effects of variables in the *q*_*t*_. For 15 min of contact, the linear effect of P concentration (with positive impact) is the most important effect, followed by the linear and quadratic effects of pH (with negative impact). As the concentration of P in solution increases, more saturated are the active sites on the NPs, leading to *q*_*t*_ increase. Consider Fig. 1, the removal of P decreases with the increase of pH and thus the amount of P on the NPs also decreases. While for 1 h of contact time, pH has no significant impact on the response, after 24 h, it has negative impact.

Figure 2 illustrates how the interactions of the main effects influenced the response after identical contact time periods.

Figure 3SI (in Online Resource) shows that an increase in the initial concentration of P has a negative effect on the removal percentage. A maximum removal efficiency of 100% was achieved at all contact times. The minimum removal efficiencies observed were 55, 60, and 60% for 15 min, 1 h, and 24 h, respectively. For pH, a negative trend is observed for the three contact times, despite an initial increase in removal at lower pH values. This resulted in a curve that ascends to a peak at ca. pH 6 and then significantly descends, forming an arc. This is expected as the PZC of NPs is around 6.3. Phosphorus removals were 55, 65, and 25% at pH 4, 6, and 10 for 15 min; 62, 70, and 40% at pH 4, 6, and 10 for 1 h; and 80, 85, and 40% at pH 4, 5.5, and 10 for 24 h of exposure. For the sorbent dose variable, a generally positive arc-shaped trend is observed across all three contact times, characterized by an initial increase in P removal and a peak at a sorbent dose between 1.8 and 2.0 g/L, followed by a decrease for a sorbent dose exceeding 2 g/L. The P removals achieved were 25, 70, and 63% using 0.2, 2.0, and 2.5 g NPs/L for 15 min; 25, 75, and 70% using 0.2, 1.8, and 2.5 g NPs/L for 1 h; and 35, 90, and 83% using 0.2, 2.0, and 2.5 g/L for 24 h of contact time.

Consider Fig. 2, for the [P] × m/V interaction, an increase in the initial P concentration leads to a decrease in removal efficiency at all three contact times and across all sorbent doses. The lowest sorbent dose (blue line) results in significantly lower P removals compared to the other two doses (red and green lines). The removal efficiencies of the two higher doses are similar. Therefore, the use of the highest sorbent dose is not justified, making 1.35 g/L the optimal dose. In the [P]*pH interaction, as the initial P concentration increases, P removal efficiency decreases. The optimal pH condition for P removal after 15 min is pH 7, followed by pH 4 and pH 10. For longer contact times and higher P initial concentration (Fig. 2c), the removals tend to be higher at pH 4 than pH 7. Regarding m/V*pH interaction, an increase in the sorbent dose leads to higher P removals at all three contact times. However, this trend is not linear. For example, the maximum removal at 15 min is achieved using 2.1 g NPs/L at pH 7, 2.3 g NPs/L at pH 4, and 1.6 g NPs/L at pH 10. The pH 10 condition provides the lowest P removals at all contact times.

### Response surface methodology for P removal and operational parameter optimization

RSM was applied to optimize the experimental conditions and construct a quadratic model based on the responses obtained in the experimental essays. The quadratic model is a mathematical model used in RSM that describes the relationship between the independent variables (in this case, the operational parameters) and the dependent variable (P removal). To obtain the reduced models, which are simplified versions of the full quadratic model, the statistically significant factors identified through Pareto charts were employed. These reduced models only include the most influential factors, thereby simplifying the interpretation without significantly compromising the predictive accuracy.

Table 3 shows the uncoaded values of the independent variables in the reduced models obtained for the response of P removal (%). The quality of the fit between the experimental and the calculated data is indicated by the coefficient of determination (*R*^2^) and the adjusted coefficient of determination (*R*^2^_adj_). *R*^2^ is commonly used to evaluate the suitability of the model’s fit to the experimental data and to compare the fitness of different models with the same number of variables. *R*^2^_adj_ is more suitable to compare and evaluate the fit of models with different numbers of variables. The *R*^2^ values of the reduced models for P were 0.9709, 0.9339, and 0.9425, and the *R*^2^_adj_ values were 0.9418, 0.8843, and 0.8994 for 15 min, 1 h, and 24 h, respectively. These values show a good and robust fit of the models.

**Table 3:** Reduced models of the P removal (%) and the respective *R*^2^ and *R*^2^ adjusted as function of the significant variables (*p*-value < 0.05)

15 min	Reduced response model	Removal (%) = − 66.40 − 4.23 [P] + 78.20 m/V + 36.06 pH − 2.90 m/V*pH + 0.08 [P]*[P] − 15.25 m/V*m/V − 2.63 pH*pH
	*R*^2^	0.9709
	*R*^2^_adj_	0.9418
1 h	Reduced response model	Removal (%) = − 20.53 − 4.95 [P] + 69.30 m/V + 27.26 pH + 0.10 [P]*[P] − 18.62 m/V*m/V − 2.24 pH*pH
	*R*^2^	0.9339
	*R*^2^_adj_	0.8843
24 h	Reduced response model	Removal (%) = − 53.32 + 0.63 [P] + 66.38 m/V + 32.08 pH − 0.40 [P]*pH − 16.69 m/V*m/V − 2.34 pH*pH
	*R*^2^	0.9425
	*R*^2^_adj_	0.8994

Figure 3 displays the 3D plots of the P removal at different time intervals: 15 min, 1 h, and 24 h. Overall, the maximum P removals are obtained under conditions of lower initial P concentration and pH, and higher sorbent doses and contact times. Phosphorus concentration is the most influential variable having a negative impact on the response. The effect of the concentration variable is mitigated by the dose of the sorbent. For high P concentrations, between 17 and 26 mg/L, the ideal sorbent dose range is between 1.4 and 2.1 g/L. After 24 h of contact time, a higher pH has a higher impact, reducing P removal. In the pH*[P] graphs (Fig. 3c), it was evident that the impact of pH on P removal is considerably less than that of [P]. However, at elevated P concentrations, it was observed that the highest removals occur within a pH range of 4 to 8.

The optimization of operational parameters can maximize P removal. The optimized variable values from the reduced response model indicate that for P removal to exceed 90% for 5 mg P/L, the optimized conditions are a sorbent dose greater than 1.3 g/L and a pH range between 5 and 7, all within a contact time of 15 min. For a contact time of 1 and 24 h, the conditions adjust to a sorbent dose greater than 0.97 and 0.83 g/L, respectively, with the pH range remaining the same.

### Validation of the reduced response model and effect of temperature

The predictions of the experimental conditions needed to obtain P removal percentages above 90% determined by the model were confirmed experimentally. The results of the P removal percentages predicted by DoE and obtained experimentally are listed in Table 4. As can be observed, the P removal percentages achieved by the CoFe_2_O_4_ NPs were generally higher than those predicted by DOE.

**Table 4:** Removal of P (%) determined by reduced response model and obtained experimentally

Time	[P]_*i*_ (mg/L)	m/V (g/L)	pH	*R* (%)	
				Model	Experimental
15 min	5	1	7	81	91
1 h				89	92
24 h				95	93

Typically, the temperature of industrial wastewaters is higher than the ambient temperature (20 °C). Therefore, the effect of temperature on the sorption process was evaluated, as depicted in Fig. 4SI (in Online Resource). It was observed that the efficiency of the NPs does not change with the temperature of the wastewater.

### Comparison with studies from literature

Several studies have investigated the application of magnetic nanoparticles for P removal, with performance varying significantly depending on the material and operational conditions. Faranak and Masome (2021) employed a central composite design to optimize P removal from synthetic solutions, achieving 85% removal at pH 4. However, the Fe_3_O_4_/Mn_0.75_Zn_0.25_Fe_2_O_4_ NPs used exhibit significantly lower performance at neutral pH, with removal rates dropping below 15%. Additionally, their study required a high sorbent dose (16 g/L) and long contact times (3 h). Zhang et al. (2017) developed magnetic zirconium-iron oxide nanoparticles for P removal achieving high removal percentages at acidic pH (99.8% at pH 1.5), but the performance significantly decreased with increasing pH, reaching only 60–87% at neutral pH. This emphasizes the strong pH dependence of this sorbent, limiting its applicability in neutral effluents and wastewaters. Similar to Faranak and Masome (2021), the use of synthetic P solution instead of real wastewater might have influenced the observed removal efficiencies. Moreover, the extended contact time requirement (24 h) and high sorbent dosage (2 g/L) suggest a less efficient process compared to our optimized conditions. The MgO-coated magnetic Fe_3_O_4_@SiO_2_ NPs developed by Li et al. (2022) demonstrate high stability and removal efficiency across a wide pH range (3–11), achieving over 89% P removal from a 100 mg P/L monoelemental synthetic solution with a sorbent dose of 625 mg/L. The equilibrium was reached within 60 min, with a high maximum removal efficiency of 94.3%.

Our findings demonstrate high P adsorption (> 90%) at neutral pH within a short contact time (15 min) using a relatively low sorbent dose (1 g/L). This aligns with the high efficiency and fast adsorption kinetics observed in the work of Li et al. (2022), while also offering improvements in operational parameters such as shorter contact times. The exceptional performance of CoFe_2_O_4_ NPs for P removal from real wastewaters highlights their potential for practical applications, including scenarios where rapid treatment is required. Our work addresses the challenge of reducing P concentration in the wastewater from a pulp and paper mill. To the best of our knowledge, this is the first study to investigate P removal from real pulp and paper mill effluents, as previous studies have primarily focused on synthetic solutions.

## Conclusion

Although phosphorus is an essential element for all living organisms, the unregulated discharge of phosphorus-rich wastewaters has led to the eutrophication of water bodies, a pressing environmental issue. Our research aimed to address this challenge by focusing on achieving minimal phosphorus concentrations, a key objective in wastewater engineering and surface water management.

Our study has demonstrated the significant potential of cobalt ferrite nanoparticles as a viable solution for phosphorus removal in wastewater treatment, specifically in the context of *Eucalyptus* bleached kraft pulp mill effluents. Our findings reveal that over 90% phosphorus removal can be achieved from a contaminated stream with 5 mg P/L under optimal conditions, which include a sorbent dose greater than 1.3 g/L, a pH range between 5 and 7, and a contact time of 15 min. For longer contact times of 1 and 24 h, the required sorbent dose adjusts to greater than 0.97 and 0.83 g/L, respectively, while maintaining the same pH range.

This approach not only offers a sustainable and efficient strategy for phosphorus recovery from pulp mill effluents but also contributes to reducing environmental impact. As we move forward, it will be essential to continue exploring and refining this approach, taking into account the daily variability in the composition of industrial effluents and the progressively stringent environmental standards. The insights gained from this research pave the way for future studies aimed at enhancing the efficiency and sustainability of wastewater treatment processes.

## Supplementary Information

Below is the link to the electronic supplementary material.

Supplementary file1 (DOCX 729 KB)

## Data Availability

All data supporting the findings of this study are available within the paper and its Supplementary Information.
