# Supplementary material for: Application of Box-Behnken design to optimize the phosphorus removal from industrial wastewaters using magnetic nanoparticles
Source: Environ Sci Pollut Res Int. 2025 Feb 28;32(11):6804–16. doi: 10.1007/s11356-025-36152-6 (PMC11928393; doi:10.1007/s11356-025-36152-6)
Supplement: Supplementary file 1 — Supplementary file1 (DOCX 729 KB) [file 11356_2025_36152_MOESM1_ESM.docx]

**Supporting information**

Environmental Science and Pollution Research

**Application of Box-Behnken Design to optimize the phosphorus removal from industrial wastewaters using magnetic nanoparticles**

Celso E. D. Cardoso^a,b^, Joana C. Almeida ^a,b^, João Rocha ^a^, Eduarda Pereira^b*^

^a^Chemistry Department and CICECO-Aveiro Institute of Materials, University of Aveiro, Campus de Santiago, 3810-193, Aveiro, Portugal

^b^Chemistry Department and LAQV-REQUIMTE, University of Aveiro, Campus de Santiago, 3810-193, Aveiro, Portugal

**Corresponding author**

* eduper@ua.pt


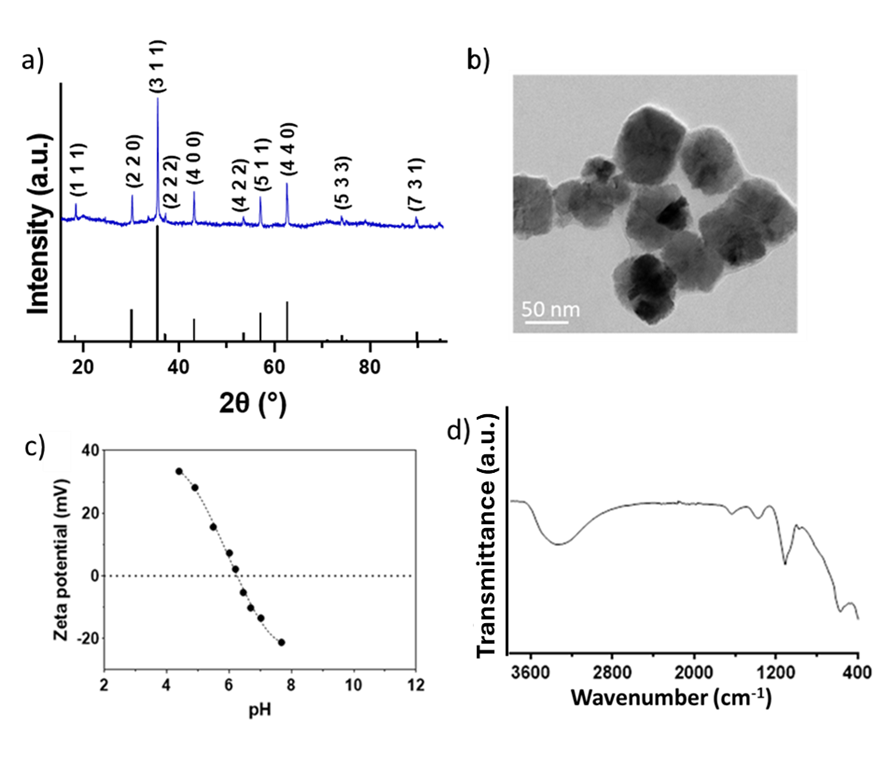


Figure 1SI – Characterization of the spinel CoFe_2_O_4_ NPs by a) Powder X-ray Diffraction; b) Transmission Electron microscopy; c) zeta potential measurements; and d) FTIR-ATR spectroscopy.


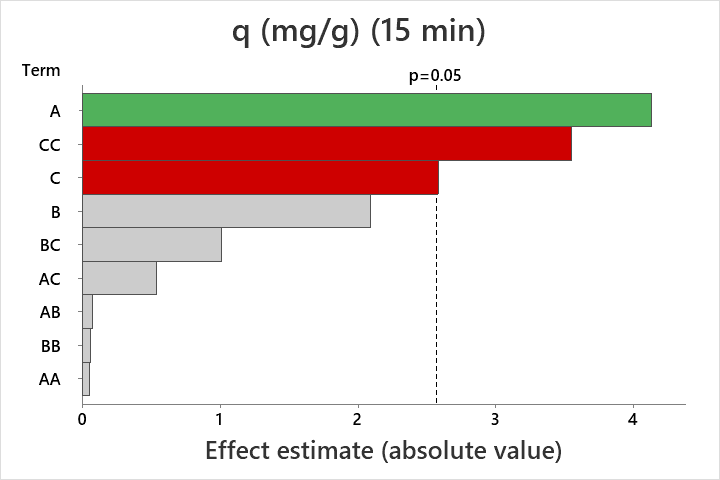

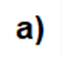

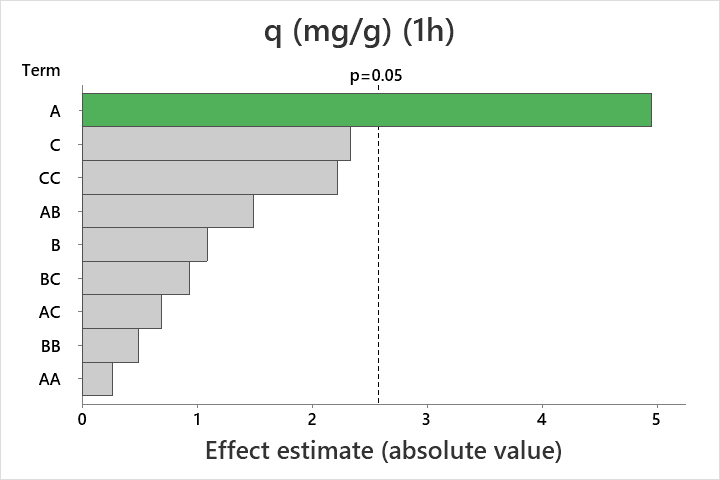

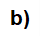

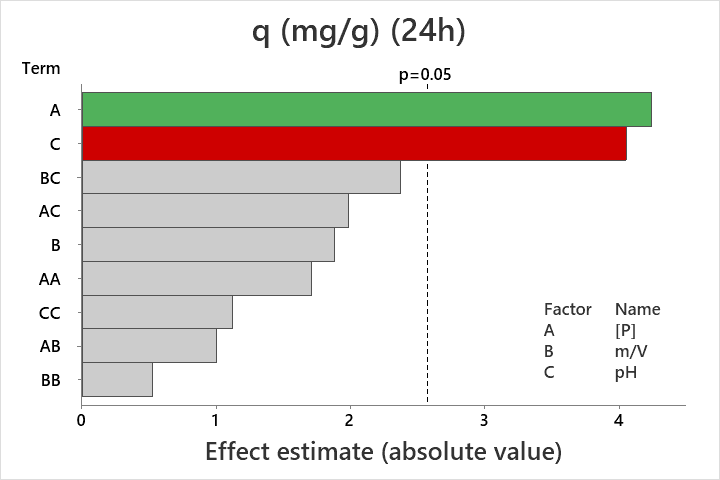

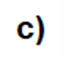


Figure 2SI – Pareto chart with the effects of the factors in the response studied (q (mg/g)) at a) 15 minutes, b) 1 hour and c) 24 hours of exposure. In the figure: A represents the initial P concentration (mg/L), B is the sorbent dose (g/L), and C is the pH. Factors with values below the dashed line are not significant.


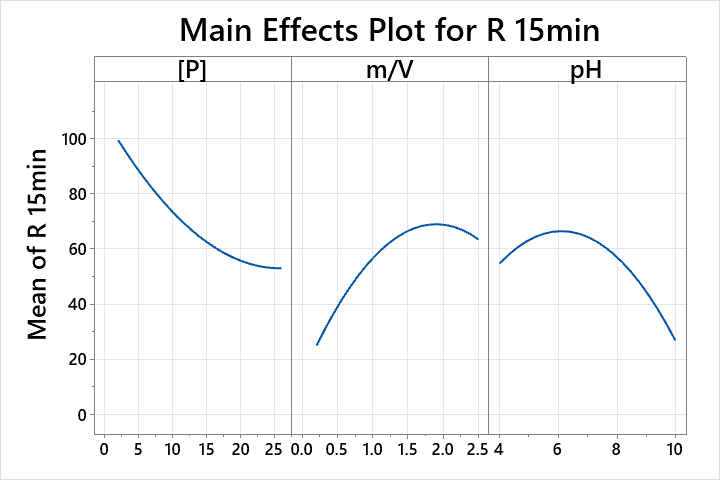

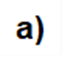

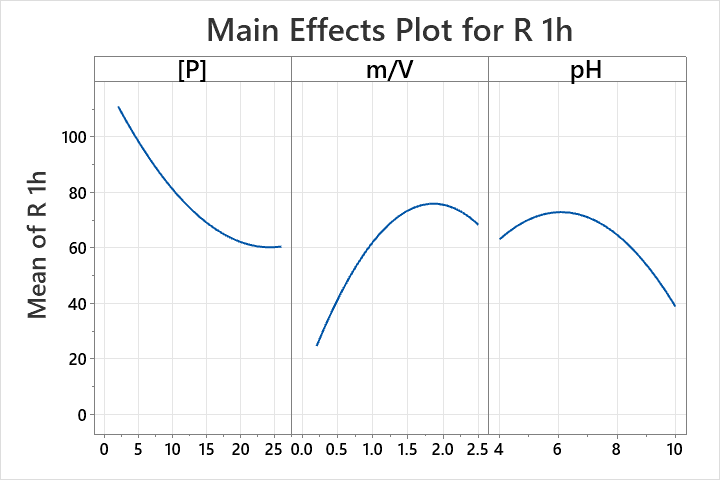

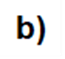

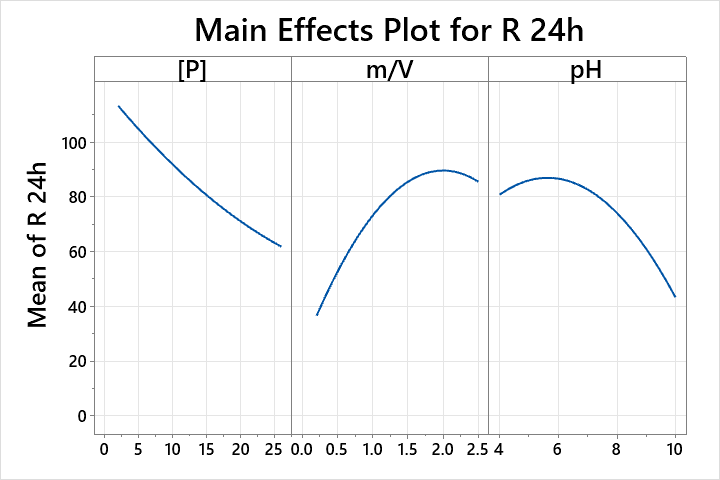

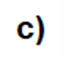


Figure 3SI – Characteristic behavior and the impact of variables main effects ([P], m/V, and pH) on the response after a) 15 minutes, b) 1 hour, and c) 24 hours of contact time.

Figure 4SI – Effect of temperature (20, 40 and 60 ºC) on the P removal efficiency from an industrial wastewater (5 mg P/L) using CoFe_2_O_4_ (1.0 g/L), at pH 7.

Table 1SI – Description of the experimental conditions according to the BBD design.

| **Experiment** | **pH** | **Dose of sorbent (g/L)** | **Initial concentration of P (mg/L)** |
| --- | --- | --- | --- |
| 1 | 7 | 0.20 | 2 |
| 2 | 7 | 0.20 | 26 |
| 3 | 7 | 2.50 | 2 |
| 4 | 7 | 2.50 | 26 |
| 5 | 4 | 1.35 | 2 |
| 6 | 4 | 1.35 | 26 |
| 7 | 10 | 1.35 | 2 |
| 8 | 10 | 1.35 | 26 |
| 9 | 4 | 0.20 | 14 |
| 10 | 4 | 2.50 | 14 |
| 11 | 10 | 0.20 | 14 |
| 12 | 10 | 2.50 | 14 |
| 13 | 7 | 1.35 | 14 |
| 14 | 7 | 1.35 | 14 |
| 15 | 7 | 1.35 | 14 |
